# *Clerodendrum inerme* Leaf Extract Alleviates Animal Behaviors, Hyperlocomotion, and Prepulse Inhibition Disruptions, Mimicking Tourette Syndrome and Schizophrenia

**DOI:** 10.1155/2012/284301

**Published:** 2012-07-15

**Authors:** Hon-Lie Chen, Hsin-Jung Lee, Wei-Jan Huang, Jui-Feng Chou, Pi-Chuan Fan, Jung-Chieh Du, Yuan-Ling Ku, Lih-Chu Chiou

**Affiliations:** ^1^Graduate Institute of Pharmacology, College of Medicine, National Taiwan University, No. 1, Jen-Ai Road, Section 1, Taipei 100, Taiwan; ^2^Department of Pharmacology, College of Medicine, National Taiwan University, No. 1, Jen-Ai Road, Section 1, Taipei 100, Taiwan; ^3^Graduate Institute of Pharmacognosy, Taipei Medical University, No. 250, Wu-Hsing Street, Taipei 110, Taiwan; ^4^Department of Pediatrics, College of Medicine, National Taiwan University, No. 1, Jen-Ai Road, Section 1, Taipei 100, Taiwan; ^5^Department of Pediatrics, Taipei City Hospital, Zhongxiao Branch, No. 87, Tongde Road, Taipei 115, Taiwan; ^6^Medical and Pharmaceutical Industry Technology and Development Center, 7F, No. 9, Wuquan Road, Wugu Dist., New Taipei 248, Taiwan; ^7^Graduate Institute of Brain and Mind Sciences, College of Medicine, National Taiwan University, No. 1, Jen-Ai Road, Section 1, Taipei 100, Taiwan

## Abstract

Previously, we found a patient with intractable motor tic disorder, a spectrum of Tourette syndrome (TS), responsive to the ground leaf juice of *Clerodendrum inerme* (*CI*). Here, we examined the effect of the ethanol extract of *CI* leaves (*CI* extract) on animal behaviors mimicking TS, hyperlocomotion, and sensorimotor gating deficit. The latter is also observed in schizophrenic patients and can be reflected by a disruption of prepulse inhibition of acoustic startle response (PPI) in animal models induced by methamphetamine and NMDA channel blockers (ketamine or MK-801), based on hyperdopaminergic and hypoglutamatergic hypotheses, respectively. *CI* extract (10–300 mg/kg, *i.p.*) dose-dependently inhibited hyperlocomotion induced by methamphetamine (2 mg/kg, *i.p.*) and PPI disruptions induced by methamphetamine, ketamine (30 mg/kg, *i.p.*), and MK-801 (0.3 mg/kg, *i.p.*) but did not affect spontaneous locomotor activity, rotarod performance, and grip force. These results suggest that *CI* extract can relieve hyperlocomotion and improve sensorimotor gating deficit, supporting the therapeutic potential of *CI* for TS and schizophrenia.

## 1. Introduction

In previous case study, we found that a 13 year-old girl who had suffered from intractable chronic motor tic disorders for more than 6 years responded dramatically to the grounded leaf juice of a local herb, *Clerodendrum inerme *(L.) Gaertn (*CI*). Her tics subsided 1 hour after taking this leaf juice. After taking the herb for 2 years, her motor tics were markedly reduced and her physical and laboratory examinations, including hemograms, liver and renal functions, blood gas, and electrolytes, were all normal [1].

The tic disorder is a neuropsychiatric disorder with a prevalence rate of 0.4–1% worldwide [2] and 5.5% in Taiwan [3], manifesting involuntary, sudden, rapid, repetitive, non-rhythmic, stereotyped movements (motor tics) or phonic tics [4]. Tourette syndrome (TS) is an idiopathic spectrum of tic disorders with multiple motor tics and at least one phonic tic lasting for at least one year and often comorbids with obsessive-compulsive disorder (OCD) and attention deficit hyperactivity disorder (ADHD) [5].

The pathogenesis of tic disorders or TS remains unclear although dopaminergic hyperreactivity in the basal ganglia and a deficit in cortico-thalamic-pallidostrial circuits have been proposed [4]. It is suggested that TS patients have overactive dopamine transporter system, resulting in reduced tonic dopamine release and upregulation of postsynaptic D2 receptors, while, upon stimulation, phasic dopamine release is increased, leading to hyperreactivity in motor responses. This hypothesis is based on the effectiveness of antipsychotic agents in clinical practices and the limited morphological studies in humans using postmortem brain tissues or clinical neuroimaging studies in patients [6, 7].

TS patients also manifest a sensorimotor gating deficit, which is believed to contribute to their premonitory urges [8] and can be reflected by an endophenotype deficit in prepulse inhibition of acoustic startle response (PPI) [9]. PPI is a neurophysiological phenomenon that the startle response to a stimulus (pulse) is inhibited if a weaker stimulus (prepulse) with the same source is preapplied within a short interval. The stimulus could be a sound, an airpuff, or a light. PPI is believed to be a processing protection in a living organism, serving as a preconscious regulator of attention, termed sensorimotor gating, to reduce the startle response that is harmful to the information professing. PPI can be assessed by the motor response in either humans (measuring the eyeblink response by electromyograph) or animals (measuring the startle jumping response) [10].

Not only in TS patients, PPI disruptions were also observed in patients with other neuropsychiatric disorders related to TS, such as ADHD [11, 12], OCD [13], and schizophrenia [14]. ADHD and OCD are two common comorbidities of TS, and TS in childhood is a risk etiology of schizophrenia [15]. Especially in schizophrenic patients, PPI disruption is believed to be a typical endophenotype of cognitive function deficits, leading to hallucination due to a flood of sensory inputs [14]. Two hypotheses of schizophrenia, hyperdopaminergic and hypoglutamatergic, have been proposed and the respective animal models have been established. The models with animals treated with dopamine mimetic agents, such as methamphetamine, which facilitates dopamine release, or apomorphine, which is a dopamine receptor agonist, are based on the hyperdopaminergic hypothesis [16]. The models with animals treated with noncompetitive NMDA channel blockers, such as phencyclidine [17], ketamine, and MK-801 [16], are based on the hypoglutamatergic hypothesis. PPI disruptions can be induced in these animal models [18–21].

So far, no available animal models perfectly mimic TS or tic disorders. Nevertheless, based on the hyperdopaminergic hypothesis, we used a mouse model treated with methamphetamine that induced hyperlocomotion to examine if the leaf extract of *CI* was effective in this model. In addition, we also examined if the *CI* leaf extract was effective in rescuing PPI disruptions induced by methamphetamine, ketamine, and MK 801.

## 2. Materials and Methods

### 2.1. Plant Material

The leaves of *CI* were collected from mangrove marshes in the riverside of southern Taiwan in March 2005. The *CI* leave sample from the same batch collection was deposited as a voucher specimen (TMU27423) in the herbarium of College of Pharmacy, Taipei Medical University.

### 2.2. Preparation of the Ethanol Extract from *CI* Leaves

The air-dried *CI* leaves (3.00 kg) were ground and repeatedly extracted with 95% ethanol (10 L) three times, each for one week. The combined ethanol layers were evaporated under reduced pressure to give a residue (246.26 g). This ethanol extract of *CI* leaves (*CI* Extract) was stored at 4°C. When used in the subsequent animal experiments, it was dissolved in dimethylsulfoxide (DMSO).

### 2.3. Animal Experiments

All animal experiments were approved by the Institutional Animal Care and Use Committee of National Taiwan University, College of Medicine (NTUMC). Male ICR mice aged 6–9 weeks were housed (3–5 mice per cage) in the holding room of NTUMC Animal Center on a 12 h/12 h reversed light schedule with free access to rodent chow and water. On the day of conducting behavioral tests, mice in their home cages were transferred to the behavioral room and acclimated for 1 hour before starting experiments. The testing chamber in each behavioral apparatus was cleaned with 70% alcohol after each mouse had completed a test session. All drugs were given by intraperitoneal (*i.p.*) injection with a volume of 0.1 mL. 

### 2.4. Locomotor Activity

The locomotor activity of the mouse was measured by its interruptions of infrared photobeams in a locomotor cage (42 cm × 42 cm × 36 cm) in the Photobeam Activity System (San Diego Instrument, San Diego, CA). After acclimation, the mouse was treated with *CI* extract or vehicle for 15 min, followed by methamphetamine (2 mg/kg). Then, the mouse was gently placed in the locomotor cage and the horizontal and vertical interruptions were counted every 5 min for 2 hours. Total locomotor activity was the sum of interruptions within 2 hours.

### 2.5. Rotarod Test

The motor coordinating activity of the mouse was measured by its performance on a rotarod in the SDI ROTOR-ROD system (San Diego Instruments, San Diego, CA). The mouse was trained four times a day for 3 days until it could stay on the rotating drum at a rotating speed of 24 rpm for at least 120 sec. Then, the mouse was subjected to the rotarod test at the rotating speed accelerating gradually from 0 to 30 rpm, and the latency to fall from the rotating drum was recorded. The cut-off time for the latency to fall was 300 sec. Animals were treated with *CI* extract or vehicle for 15 min before receiving the test.

### 2.6. Grip Strength Test

Forelimb grip strength was measured by the grip force of forepaws of the mouse using the SDI Grip Strength System (San Diego Instrument, San Diego, CA). The grip strength was recorded by the maximum of three permissible readings (in grams). The test was conducted every 2 min for three times, and the averaged grip strength was recorded. Animals were treated with *CI* extract or vehicle for 15 min before the test.

### 2.7. PPI Test

The PPI test was conducted with a PPI apparatus (SR-LAB, San Diego Instruments, San Diego, CA) consisting of a startle chamber equipped with various programming acoustic stimulations. After acclimation, the mouse was gently placed in the startle chamber for a 4 min acclimation period with a background noise of 65 dB, which continued throughout the whole PPI test session. One PPI test session consisted of 4 types of startle trials, including the trial with the startle pulse (115 dB) alone (PULSEALONE; 115 dB, 20 ms), two trials with the startle pulse paired with 71 and 77 dB prepulses, respectively (PREPULSE + PULSE; 71 dB + 115 dB and 77 dB + 115 dB), and the trial without stimulus (NOSTIM; background 65 dB only). A test session started and ended, respectively, with four NOSTIM trials and four PULSEALONE trials. In between, each of the four types of trials was presented 14 times randomly, that is, 56 trials were given in a test session. The intertrial interval was given randomly from 5 to 20 s. In the PREPULSE + PULSE trial, a 71 or 77 dB prepulse was given 120 ms before the 115 dB-pulse. The magnitude of PPI (PPI%) was determined, after summarizing the startle responses in PULSEALONE and PREPULSE + PULSE trials, according to the equation (PULSEALON − PREPULSE + PULSE)/PULSEALONE × 100%. *CI* extract or vehicle was given to the animals for 15 min, followed by methamphetamine (2 mg/kg), ketamine (30 mg/kg), or MK-801 (0.3 mg/kg). The PPI test was conducted 10 min after injection of methamphetamine or ketamine and 20 min after MK-801 injection.

### 2.8. Chemicals

Methamphetamine was purchased from Sigma-Aldrich (St. Louis, MO) and ketamine was purchased from Parke-Davis (Taoyuan, Taiwan) with the approval of Food and Drug Administration, Department of Health, Executive Yuan, Taiwan. MK-801, haloperidol, and clozapine were purchased from Sigma-Aldrich. *CI* extract, haloperidol, and clozapine were dissolved in DMSO. Methamphetamine, ketamine, and MK-801 were dissolved in normal saline.

### 2.9. Statistics

Data were expressed as the mean ± S.E.M. Statistical comparisons among groups were analyzed by ANOVA with Tukey *post hoc* test, and differences between groups were analyzed by Student's *t-*test. Two-way ANOVA with Bonferroni's *post hoc* test was used to analyze differences in the time courses of locomotor activity among groups. Differences were considered significant if *P* < 0.05.

## 3. Results and Discussion

### 3.1. Effects of *CI* Extract on Motor Functions

#### 3.1.1. *CI* Extract Reduced Methamphetamine-Induced Hyperlocomotion at Doses without Affecting Motor Coordination and Muscle Power

**Figure 1:**
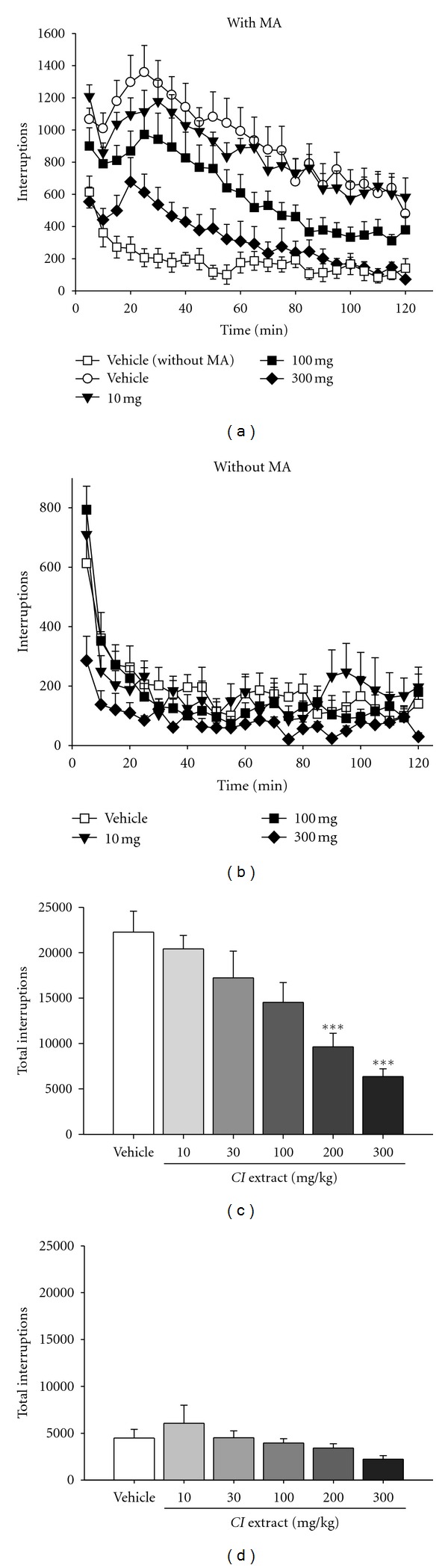
*Cl* extract dose-dependently inhibited hyperlocomotor activity induced by methamphetamine but not spontaneous locomotor activity. Effects of *CI* extract on the locomotor activity of mice with (a and c) or without (b and d) methamphetamine (MA) treatment. The locomotor activity was measured by horizontal and vertical interruptions of infrared (IR) beams in the testing chamber every 5 min for 2 hours after *i.p.* injection of 2 mg/kg methamphetamine (a) and (c) or normal saline (b) and (d). Mice were pretreated with* CI* extract (10–300 mg/kg,* i.p.*) or vehicle for 15 min before the test. (a) and (b): Time courses of changes of IR-beam interruptions after injection of methamphetamine (a) or normal saline (b) in groups pretreated with vehicle or various doses of *CI* extract. (a): For comparison, the vehicle group without MA pretreatment (open squares) was also included. Two-way ANOVA for MA-treated groups with repeated measures over time analysis indicated significant differences with a main effect of Treatment (*F*_3,621_ =158.2; *P* <0.05) and Time (*F*_23,621_ =15.63; *P* < 0.05), and in the interaction of Treatment by Time (*F*_69,621_ = 0.34; *P* < 0.05). (b): There was no significant difference among groups (two-way ANOVA). (c) and (d): Total interruptions in 2 hours after MA or saline treatment in various groups. ***P < 0.001 versus the vehicle group (*F*_5,48_ = 9.591; *P* < 0.001 for (c); *F*_5,48_ = 2.045, *P* = 0.086 for (d), one-way ANOVA with *post hoc* Tukey test). *N* = 9.

In normal saline-treated mice, the spontaneous locomotor activity was markedly decreased within 10–15 min after they were placed in the locomotor cage due to acclimation (open squares in Figures 1(a) and 1(b)). In contrast, in mice treated with 2 mg/kg (*i.p.*) of methamphetamine, the locomotor activity was significantly increased. This hyperlocomotion, after a transient decrease, reached a peak at 20–30 min and declined gradually within 2 hours (open circles in Figure 1(a)). The total locomotor activity within 2 hours in methamphetamine-treated mice was significantly higher than that in the group without methamphetamine treatment (22327 ± 2309 versus 4549 ± 926 interruptions, *P* < 0.001) (open bars in Figures 1(b) and 1(d)).

Methamphetamine-induced hyperlocomotion was significantly decreased in the group pretreated with *i.p.* injection of *CI* Extract for 15 min, as compared with the vehicle-pretreated group. This effect of *CI* extract was dose-dependent at tested doses ranging from 10 to 300 mg/kg (Figures 1(a) and 1(c)). On the other hand, in mice without treatment with methamphetamine, *CI* extract did not alter spontaneous locomotor activity (Figures 1(b) and 1(d)).

#### 3.1.2. *CI* Extract Did Not Affect the Rotarod Performance and Grip Strength

**Figure 2:**
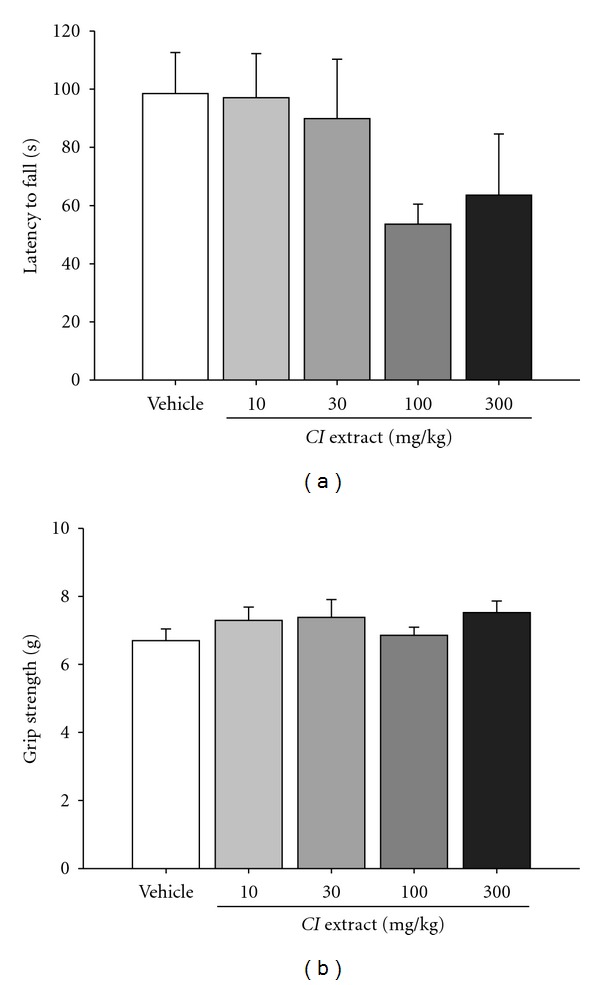
*CI* extract had no effect on motor coordination and muscle power. (a): Effects of *CI* extract on the motor coordination measured by the latency to fall in the rotarod test in mice. The mouse was pretrained until the latency to fall was greater than 120 sec. (b): Effects of *CI* extract on the grip strength (muscle power) of mice. The grip strength of forepaws of the mouse was measured by a grip strength meter three times every 2 min and averaged. Mice were pretreated with *CI* extract and vehicle for 15 min before the tests. No significant difference was found among groups (*F*_4,40_ = 1.590, *P* = 0.196 for (a); *F*_4,40_ = 0.897, *P* = 0.475 for (b), one-way ANOVA). *N* = *9*.

We further examined the effects of *CI* extract on other motor functions, including the motor coordination and muscle power, which were measured by the rotarod performance and grip strength, respectively. In the rotarod test, the performance in mice treated with *CI* extract at the dose up to 300 mg/kg was not significantly different from that in vehicle-treated mice (Figure 2(a)). The grip strength, a measurement for muscle contraction power, was also unaffected by *CI* extract at the dose up to 300 mg/kg (Figure 2(b)).

These results indicate that the ethanol extract of *CI* leaves inhibited methamphetamine-induced hyperlocomotion at doses without affecting spontaneous locomotor activity or other motor functions, such as motor coordination and muscle power.

### 3.2. Effect of *CI* Extract on PPI Disruptions

#### 3.2.1. Establishing a PPI Disruption Model Induced by Methamphetamine

**Figure 3:**
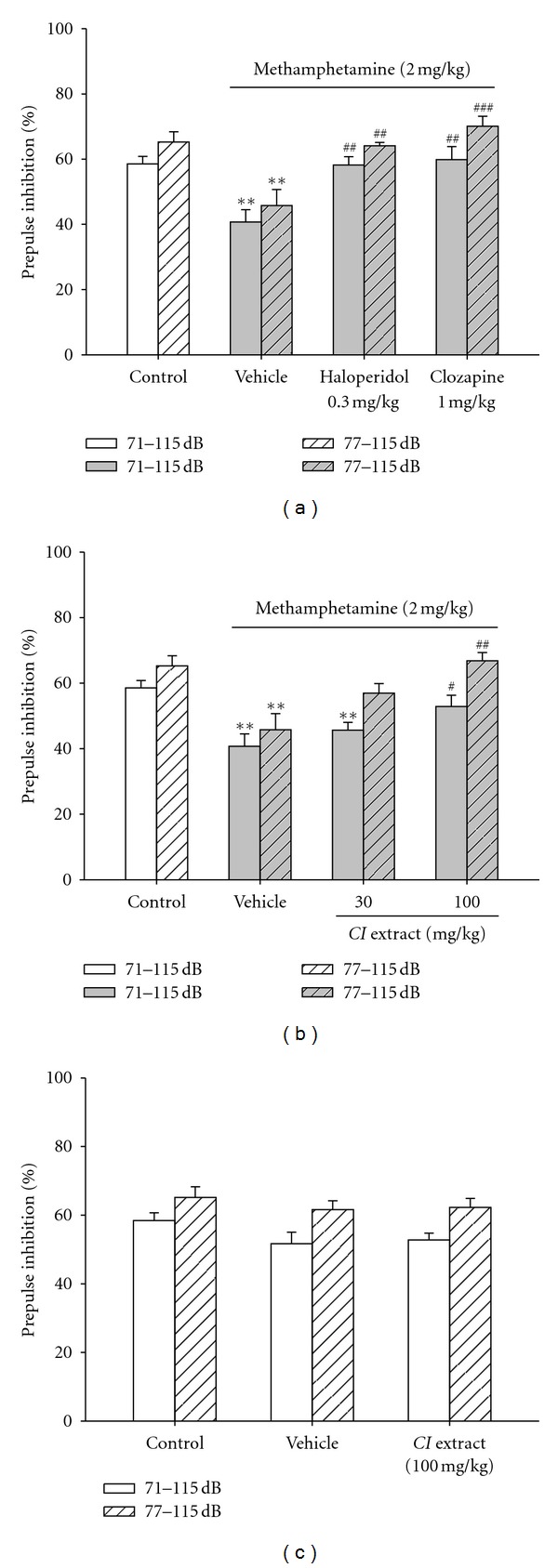
*CI* extract, like antipsychotics, prevented methamphetamine-induced disruption of prepulse inhibition of acoustic startle response (PPI) in mice. (a): Effects of haloperidol and clozapine, typical and atypical antipsychotic agents, respectively, on the PPI disruption induced by methamphetamine. (b): Effects of *CI* extract on the PPI disruption induced by methamphetamine. The magnitude of PPI in the startle response to a 115 dB acoustic stimulation paired with a prepulse of 71 dB (71–115 dB) or 77 dB (77–115 dB) 120 ms ahead was measured as described in Section 2. Mice were pretreated with haloperidol (0.3 mg/kg, *i.p.*), clozapine (1 mg/kg, *i.p.*), *CI* extract (30 or 100 mg/kg, *i.p.*), or vehicle (*i.p.*) for 15 min, followed by methamphetamine (2 mg/kg, *i.p.*) for 10 min before the test. (c): The effect of *CI* Extract on PPI in mice without methamphetamine treatment. Normal mice were treated with *CI* extract (100 mg/kg, *i.p.*) or vehicle (*i.p.*) for 15 min before being subjected to the PPI test. The control group is the mice treated with normal saline only (without methamphetamine). **P < 0.01 versus the control group; ^#^ *P* < 0.05, ^##^ *P* < 0.01, and ^###^ *P* < 0.001 versus the vehicle group with the same acoustic stimulation protocol, 71–115 dB or 77–115 dB (Student's* t*-test). *N* = 8.

In addition to tic attacks, TS patients also manifest a deficit in sensorimotor gating function, which can be measured by a disruption of PPI in humans or animal models. Increasing dopaminergic activity has been reported to disrupt PPI, and this disruption can be blocked by antipsychotic agents [22], which are also effective antitic drugs clinically. We, therefore, established a PPI disruption model in mice induced by *i.p*. injection of methamphetamine (2 mg/kg), which increases dopamine release. The startle response to a 115 dB acoustic stimulation in normal saline-treated mice was inhibited by a prepulse acoustic stimulation at 71 dB by 58.9 ± 2.3%, that is, the PPI magnitude in this 71–115 dB protocol is 58.9 ± 2.3% (open bar in Figure 3(a)). Increasing the prepulse acoustic stimulation to 77 dB, the magnitude of PPI was increased to 65.6 ± 3.1% (open slashed bar in Figure 3(a)). In animals treated with methamphetamine (2 mg/kg, *i.p.*), PPI was significantly reduced to 41.1 ± 3.8% and 46.1 ± 4.9%, respectively, in response to 71–115 dB and 77–115 dB stimulations. In mice pretreated with the antipsychotic agent, either a typical one (haloperidol, 0.3 mg/kg, *i.p.*) or an atypical one (clozapine, 1 mg/kg, *i.p.*) for 15 min, PPI was significantly restored to the levels of control groups in both 71–115 dB and 77–115 dB protocols (Figure 3(a)). Therefore, a PPI disruption model in mice was established by *i.p.* injection of 2 mg/kg methamphetamine, and this disruption can be prevented by typical and atypical antipsychotics.

#### 3.2.2. *CI* Extract Prevented Methamphetamine-Disrupted PPI

Effects of *CI* extract (30 or 100 mg/kg,* i.p.*) on methamphetamine-disrupted PPI were then examined. In mice pretreated with *CI* extract for 15 min, the magnitudes of PPI in response to 71–115 dB and 77–115 dB stimulations, respectively, were restored to 46.0 ± 2.3% and 57.3 ± 2.9% by 30 mg/kg *CI* extract and to 53.2 ± 3.4% and 67.2 ± 2.5% by 100 mg/kg *CI* extract, which were not significantly different from the control groups (Figure 3(b)).

In mice without treatment with methamphetamine, *CI* Extract did not affect PPI (Figure 3(c)). Therefore, *CI* Extract is effective in protecting mice from the PPI disruption induced by methamphetamine at an optimal dose of 100 mg/kg by *i.p.* injection, but not the PPI in normal mice.

#### 3.2.3. *CI* Extract Prevented PPI Disruptions Induced by Ketamine

**Figure 4:**
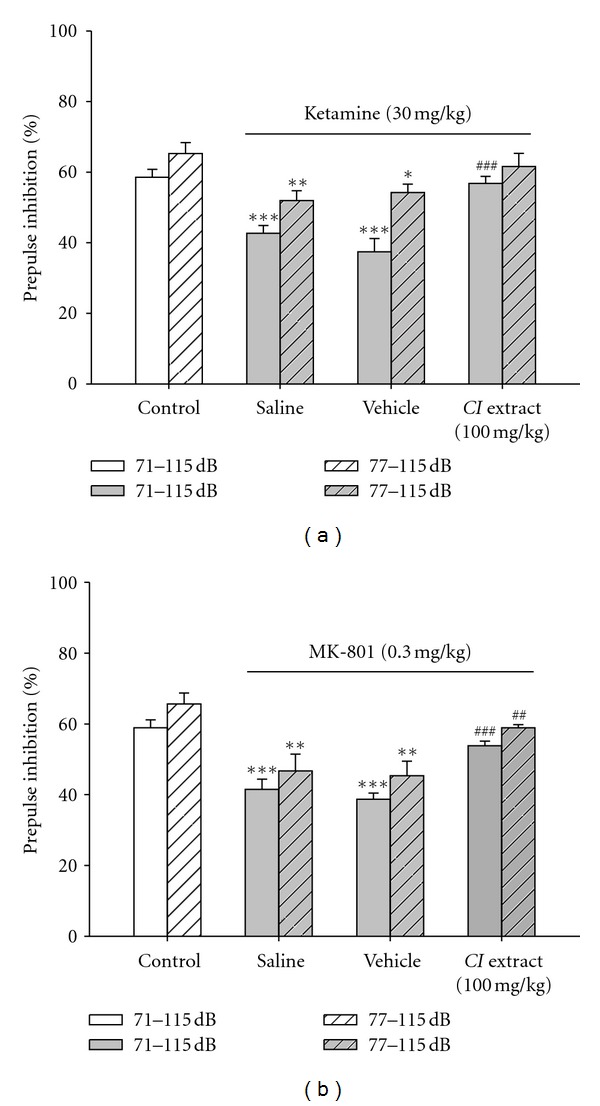
*CI* extract prevented PPI disruptions induced by NMDA channel blockers in mice. Effects of *CI* extract on PPI disruptions induced by NMDA channel blockers, ketamine (a) and MK-801(b). Mice were pretreated with *CI* extract (100 mg/kg, *i.p.*) or vehicle for 15 min, followed by ketamine (30 mg/kg, *i.p.*) for 10 min (a) or MK-801 (0.3 mg/kg, *i.p.*) for 20 min (b). The control group is the mice treated with normal saline alone (without NMDA blockers). The saline group is the mice pretreated with normal saline before being challenged with ketamine or MK-801. *P < 0.05, **P < 0.01, and ***P < 0.001 versus the control group; ^##^ *P* < 0.01 and ^###^ *P* < 0.001 versus the vehicle group with the same acoustic stimulation protocol (Student's* t* test). *N* = 8.

In addition to dopamine mimetic agents, PPI disruptions can also be induced by noncompetitive NMDA channel blockers, such as ketamine or MK-801 [10, 19, 22]. We, therefore, also examined if *CI* extract was effective in these PPI disruption models. In mice injected with ketamine (30 mg/kg, *i.p.*) for 10 min, the magnitude of PPI in response to 71–115 dB stimulation was decreased from 58.9%  ± 2.3% to 43.0%  ± 2.2%. Increasing the prepulse to 77 dB, PPI was decreased from 65.6%  ± 3.1% to 52.3%  ± 2.8% (Figure 4(a)). The PPI disruption induced by ketamine (30 mg/kg,* i.p.*) was not different from that induced by methamphetamine (2 mg/kg,* i.p.*) (Figure 4(a) versus Figure 3, *P *= 0.662 for 71–115 dB and *P *= 0.292 for 77–115 dB protocols).

As in the PPI disruption model induced by methamphetamine, *CI* extract (100 mg/kg, *i.p*.) also prevented the PPI disruption induced by ketamine. The magnitudes of PPI in response to 71–115 dB and 77–115 dB stimulations were restored by *CI* extract to 57.2%  ± 2.0% and 61.9%  ± 3.7%, respectively, which were similar to control levels (Figure 4(a)).

#### 3.2.4. *CI* Extract Prevented PPI Disruptions Induced by MK-801

In mice receiving *i.p.* injection of 0.3 mg/kg MK-801 for 15 min, the magnitude of PPI in response to a 71 dB prepulse acoustic stimulation was decreased from 58.9%  ± 2.3% to 41.5%  ± 2.9%. Increasing the prepulse stimulus to 77 dB, the PPI was decreased from 65.6%  ± 3.1% to 46.7%  ± 4.8% (Figure 4(b)). We, therefore, also successfully established a PPI disruption model induced by *i.p.* injection of MK-801. The PPI disruption induced by MK-801 (Figure 4(b)) was not significantly different from that by ketamine (Figure 4(a), *P* = 0.676, 71–115 dB and *P* = 0.328, 77–115 dB) or by methamphetamine (Figure 3, *P* = 0.933, 71–115 dB and *P* = 0.930, 77–115 dB). As in the PPI disruption model induced by methamphetamine or ketamine, *CI* extract (100 mg/kg, *i.p*.) also prevented the PPI disruption induced by MK-801 (Figure 4(b)). The magnitudes of PPI induced by 71–115 dB and 77–115 dB stimulations were restored by *CI* extract to 53.8%  ± 1.3% and 58.9%  ± 0.9%, respectively, which were similar to control levels.

#### 3.2.5. Clinical Therapeutic Potential of *CI*

The patient in our case report [1] continues to visit our pediatric neurology clinic for her seizure control. On her latest visit to our clinic, being 6 years after she took *CI*, she reported that she had almost no more motor tics, which, when occurred occasionally, would subside 1 hour after taking *CI*. This argues against the possibility that her tic syndromes were naturally remitted with more matured brain circuits after growing up [4]. Given that antipsychotics were ineffective in this case, we suggest that the action mechanism(s) of *CI* is other than directly blocking dopaminergic receptors. This inference is also supported by the effectiveness of *CI* extract in rescuing the PPI disruptions induced by noncompetitive NMDA channel blockers (ketamine and MK-801), for which dopamine receptor blockers are ineffective [16, 22]. However, the possibility that *CI* indirectly increases dopaminergic activity in the prefrontal cortex by increasing dopamine release or inhibiting dopamine degradation, such as catecholamine-O-methyl-transferase, cannot be excluded. Identification of the active constituent(s) in this ethanol extract of *CI* leaves is undergoing in our laboratory and will be helpful in revealing the underline mechanism(s) of action of this herb.

## 4. Conclusions

In summary, we found that *CI* extract inhibited methamphetamine-induced hyperlocomotion and PPI disruptions induced by methamphetamine, ketamine, and MK-801 at doses without affecting the spontaneous locomotor activity, rotarod performance, and grip force in mice. These findings suggest that *CI* extract can relieve hyperlocomotion and improve sensorimotor gating deficit. The latter effect may prevent the urge of tic attacks in TS patients and be helpful for other psychiatric disorders such as schizophrenia, ADHD, and OCD.
